# Pandemic delay: social implications and challenges for palliative care

**DOI:** 10.1177/26323524231159146

**Published:** 2023-03-21

**Authors:** Emma Kirby, John I MacArtney

**Affiliations:** Centre for Social Research in Health, University of New South Wales, John Goodsell Building, Kensington, NSW 2052, Australia. Unit of Academic Primary Care, Division of Health Sciences, Warwick Medical School, University of Warwick, Coventry, UK; Unit of Academic Primary Care, Division of Health Sciences, Warwick Medical School, University of Warwick, Coventry, UK

The COVID-19 pandemic has profoundly affected the provision of palliative care, as it has health services and systems more broadly. Aside from the morbidity and mortality caused by the severe acute respiratory syndrome coronavirus 2 (SARS-CoV-2) itself, the wider pandemic implications continue to be felt in manifold ways. Research and practice responses to the early waves of COVID-19 focused on measures to mitigate and control the spread of the virus including modelling related to healthcare capacity, public health measures, provision of personal protective equipment, and vaccine development. However, more recent attention has turned to longer-term implications of these measures and ‘living with COVID’, including *The Lancet Oncology Commission*’s report into the unintended consequences for cancer diagnosis, care, and treatment.^1^ As we approach the 3-year milestone since the onset of COVID-19, we enter a period when the effects of pandemic-related protections and containment measures become noticeably more visible. This is especially true in circumstances where patients, their families, and their health professionals, are grappling with the effects of what have been called *pandemic delays*.

The term pandemic delay brings together several broad, but related, issues and concerns associated with the effects of the pandemic on healthcare processes, systems, and people. Responses to COVID-19 waves saw reductions in screening and testing, cancellations or postponement of surgeries, and other treatment delays.^2^ In addition, various factors have been suggested to describe how or explain why COVID-19 and related protections have affected healthcare systems, processes, practices, and experiences of timely diagnosis or treatment. These include concerns about waiting times, transmission of COVID-19 in health settings, ongoing health service restrictions, and interpreting symptoms as not significant enough to ‘take up space’ in already-overloaded healthcare services.^3^ To better understand these effects, existing models and language around diagnosis delay and help-seeking are being repurposed to understand experiences of delay during the first 3 years of the pandemic.^1^ What has had less attention is how the social, cultural, and political context affects, and has been affected by, changes to diagnostic processes and palliative provision.

## How are forms of pandemic delay understood?

The impact of COVID-19 and associated containment-related protections upon healthcare and diagnostic processes, as well as morbidity and mortality, have been explored under several conceptual and theoretic rubrics seeking to describe and explain delays in diagnosis. The pandemic has brought predictions of palliative care patient ‘surges’, of increasing numbers of ‘avoidable deaths’, and of changes in the clinical and demographic profiles of patients who require palliative care.^1,4^ Research has already highlighted reductions in urgent referrals for suspected cancer during 2020, related to significantly less patients consulting with general practiotioners (GPs).^5^ Fewer presentations to primary care, and thus fewer referrals and diagnosis, have been reported as consequences of lockdowns and other pandemic-driven non-pharmaceutical interventions.^6^ Fewer diagnoses in this period may mean that patients are presenting *later*, with more advanced disease, and the potential for poorer prognosis.^2,7^ Patterns of diagnostic delay have prompted modelling that predicts increases in the number of deaths up to 5 years after diagnosis compared with pre-pandemic figures.^8^ This has led to calls for the development of new strategies and pathways to and through diagnosis and subsequent treatment, to palliative care.^6^

Discussions of pandemic delay are therefore bringing together two healthcare literatures that often sit at either end of the patient journey: (early) diagnosis and transitions to palliative care. This is because delays in screening, presentation, diagnosis, or treatment, are likely to increase the complexity of palliative care needs for many patients. Moreover, trends towards late(r) diagnosis may prompt additional need for emergency or rapid integration of palliative care, posing new challenges for health services. Indeed, palliative care services internationally have already experienced considerable change during COVID-19. Renewed attention has advanced health-system awareness of ensuring a continuum of care, of timely palliative care integration, and of discussion on what matters for people nearing the end of life. Service providers have reflexively and iteratively responded to fluctuating numbers of patients, new demands on resources, and unfolding requirements for practice and quality of care.^9,10^ Significant changes to palliative services, accelerating nascent initiatives locally and nationally,^9^ have rapidly developed and expanded (specialist) community palliative services such as hospice-at-home;^11^ re-emphasised the crucial role of primary care and its integration into community palliative care provision;^12^ and highlighted how established practices, such as Advanced (or Anticipatory) Care Planning (ACP) will need to adapt to the new challenges of an often dispersed, remote, untimely, and uncertain context of palliative care delivery.^13^ We turn our attention here to exploring the normative underpinnings of this convergent field of delayed diagnosis and palliative care practices, to foreground some of the social and cultural issues in the emerging discourse around pandemic delay.

## What social and cultural issues are shaping pandemic delay?

Previous sociological and anthropological work has productively complicated the interpretation of notions of ‘patient delay’ by highlighting how symptoms and bodily sensations and related processes, are interpreted within a social and cultural context that affects interpretations of what is timely.^14^ Patients are then encouraged via practices of shared decision-making to invoke their subjective, embodied, and cultural choices, which the GP should include in assessing what action to take and when.^15^ Evidence is starting to emerge that reveals how pandemic protections, fears, and cultural imaginaries related to viral transmission and danger shaped interpretations of risky symptoms and affected how and when patients presented to primary care.^3,7^ COVID-19 has prompted a reconfiguration of several forms of ambivalence: not only relating to how symptoms are experienced as needing attention, when and from whom; but to views of healthcare settings (e.g. primary care and oncology units) as risky places to be avoided until absolutely necessary,^3^ affecting how and when testing could and should be undertaken. In this way, identifying how to widen the ‘Goldilocks Zone’ within which people feel the conditions are ‘just right’ to consult a healthcare provider with a possible problem is key.^16^

The social and medical ambivalences that COVID-19 contributes to diagnostic models and practices are likely to affect ways of experiencing dying and practicing palliative care, especially for those whose diagnosis was delayed and involved late(r) stage or a terminal prognosis. Against the backdrop of pandemic-exacerbated scarcity of resources, COVID-19 has resulted in disruption to the narratives of diagnosis and palliative care. Such disruptions, to the very ways we think about and experience illness, dying, and bereavement, may be significantly reshaping the ways that palliative care is experienced and understood. This is particularly pronounced in the ways that compromises in care – such as delaying care and waiting – were experienced as emotionally and practically *necessary* at the time.^17^

## Towards a social understanding of pandemic delay

Throughout the pandemic, there have been calls to ‘follow the science’; yet, lessons from social sciences and medical humanities have been less visible at best, at worst neglected, within discussions of policy and practice.^18^ Opportunities have been missed to anticipate the ways COVID-19, and pandemic policies could exacerbate socio-economic and ethnic healthcare disparities, or how ‘post-pandemic’ public health policies may entrench – rather than challenge – existing discourses of vulnerability and marginalisation.^19^ In response, we argue here for more consideration of social issues and approaches, when examining the ongoing impact that COVID-19 is having on both the healthcare systems and the people within them. Such approaches will help us identify some of the social-political assumptions within discussions of how COVID-19 and the pandemic protections, as well as provide a more informed basis from which to explore how COVID can be understood to be contributing to the increased numbers of people diagnosed with a terminal illness.

The effects of COVID-19 on delayed terminal diagnosis are likely to be more pronounced for certain groups, particularly those already marginalised from accessing specialist palliative care (e.g. ethnic minorities, low socio-economic status, and those with non-cancer diagnosis). Responses to pandemic delays must therefore include reviewed, renewed, and revised strategies to reduce existing inequities in palliative care provision.^19^ It is also important to acknowledge that the increased emphasis on the role of palliative care in the community means that those diagnosed with a terminal illness during the last 3 years will be cared for within a discipline and service that is currently exploring its own identity. In particular, there is a strong movement within palliative care to de-medicalise dying and, instead, draw on a public health approach that includes a social model of disease.^20^ For many within this movement, the pandemic presented a moment when dying and death were socially foregrounded, and so provided a context that could prove to be a catalyst to achieve a long-held goal of developing a ‘culture to openly acknowledge death and dying’.^20^ More broadly, community palliative care approaches seek a paradigmatic shift away from dying being sequestered within hospitals and hospices, managed by healthcare professionals, towards an approach that recognises and emphasises the roles that community, family, and home have when caring for the dying. Such approaches may go some way to improving visibility for those disproportionately affected by pandemic delays.

## Conclusion

Those experiencing a pandemic-delayed terminal diagnosis and those who care for them are living and dying in medically and narratively unprecedented times. The pandemic has shifted perspectives and understandings of patterns of mortality and end-of-life experiences. What is emerging are various conceptualisations of pandemic delay through which to understand these experiences. More work is needed to improve understandings of how such experiences have shifted the normative assumptions in models of delay, access to information, knowledge, and treatment, is also urgently needed. So is attention on how the socio-cultural context of the first years of the pandemic is invoked and the ongoing politics of ‘post-pandemic’ public health policy. Examination of these significantly novel contexts – within which help is sought, diagnoses are made, and care is provided – will generate important insights that can help further improve service responses in times of crisis.

## Declarations

**Ethics approval and consent to participate:** Not applicable.

**Consent for publication:** Not applicable.

**Availability of data and materials:** Not applicable.
